# Supplementary material for: Collaborative intelligence in AI: Evaluating the performance of a council of AIs on the USMLE
Source: PLOS Digit Health. 2025 Oct 9;4(10):e0000787. doi: 10.1371/journal.pdig.0000787 (PMC12510544; doi:10.1371/journal.pdig.0000787)
Supplement: S2 File — (ZIP) [file pdig.0000787.s002.zip › S2 Data Raw data files containing unprocessed question inputs and LLM outputs including transcripts of deliberations/STEP3_Question-40-1_Type-MC-NJ.docx]

**Facilitator** **[Round 1]:** A 67-year-old man, who has been recovering in the hospital following surgical repair of a contained ruptured abdominal aortic aneurysm 10 days ago, suddenly develops severe abdominal pain. He does not have chest pain or shortness of breath. The patient's postoperative course had been uncomplicated until 4 days ago, when he developed a low-grade fever. He has been unable to tolerate a full liquid diet during the past 4 days. Medical history is remarkable for hypertension, hypercholesterolemia, and a myocardial infarction 6 years ago. Regular medications include metoprolol, lovastatin, and an 81-mg aspirin daily. He has smoked one pack of cigarettes daily for the past 40 years, and he typically drinks three to five beers daily. Vital signs now are temperature 38.1°C (100.6°F), pulse 68/min and regular, respirations 16/min, and blood pressure 150/90 mm Hg. Physical examination shows bilateral cataracts. Auscultation of the chest discloses diffuse mild wheezes and a grade 1/6 soft systolic ejection murmur at the cardiac apex. Palpation of the abdomen discloses mild to moderate mid and right upper quadrant abdominal tenderness. There is no guarding, rebound, or palpable mass. Rectal examination shows no abnormalities. Peripheral pulses are normal. Neurologic examination discloses no abnormalities. Serum electrolyte concentrations are within the reference ranges. Leukocyte count is 15,800/mm³ with mild left shift. Urinalysis shows no abnormalities. ECG shows an old inferior myocardial infarction. Chest x-ray shows scarring at the left and right cardiac bases; no masses or infiltrates are present. Which of the following is the most appropriate additional diagnostic study? (A) Arteriography of the abdomen (B) Blood cultures (C) MRI of the abdomen (D) Ultrasonography of the abdomen (E) No additional studies are needed

**AI_D:** RESTATING THE QUESTION: This case presents a 67-year-old male patient who is recovering from a surgical repair of a contained ruptured abdominal aortic aneurysm. He has suddenly started experiencing severe abdominal pain and has been unable to tolerate a full liquid diet for the past 4 days. He also developed a low-grade fever. No abnormalities are identified from a physical examination, ECG, chest x-ray, urinalysis, serum electrolyte concentrations, and peripheral pulse evaluation. His leukocyte count is elevated, indicative of an infection. Various testing modalities are being considered to determine the source of these symptoms. PLANNING: Upon restating the case, several assumptions can be generated. One assumption is that the patient's abdominal pain reflects an abdominal pathology, possibly related to the recent surgery. A second assumption may be that the fever and elevated leukocyte count indicate an infection, potentially an abdominal infection due to the surgical history. Another assumption could be that the inability to tolerate liquids might imply a digestive or gastrointestinal problem. Given these considerations, abdominal imaging seems to be a reasonable approach in this case. Despite the several approaches to abdominal imaging available, they should be evaluated based on appropriateness and specificity in relation to the given clinical situation and patient history, including the recent surgical intervention. CLINICAL REASONING: Abdominal pain in a postoperative patient is a concerning finding, especially in the context of a low-grade fever, elevated white cell count, and intolerance to liquids. These symptoms might be suggestive of an underlying infection, particularly a post-surgical intra-abdominal complication such as an abscess. It is important to confirm or rule out this consideration in a timely manner due to its potential severity. Ultrasonography is a non-invasive, readily available and reliable method for the identification of many abdominal abnormalities, including intra-abdominal abscesses. CONCLUDING: Depending on the location and size of a potential abscess, it might not create a palpable mass, and there might not be signs of peritoneal irritation such as guarding or rebound tenderness. In the given case, the patient's abdominal symptoms after recent surgery, alongside elevated leukocyte count and mild fever, seem to suggest an infectious process with a probable intra-abdominal location. Therefore, investigating for a possible intra-abdominal abscess seems justifiable. RESPONSE: Thus, the most appropriate additional diagnostic study in this situation would be Ultrasonography of the abdomen (D).

**AI_A:** RESTATING THE QUESTION: We're asked to decide the next appropriate diagnostic step for a 67-year-old man who has been recovering from a surgical repair of a ruptured abdominal aortic aneurysm. The patient is now experiencing severe abdominal pain without chest pain or shortness of breath. His postoperative course was uneventful, until four days back when he developed a fever. His significant medical history includes hypertension, hypercholesterolemia, and previous myocardial infarction, and he also has a history of smoking and alcohol use. His current physical examination shows mild to moderate abdominal tenderness without any guarding or rebound. All his labs are within normal limits except for a high leukocyte count. PLANNING: Our main assumption here is that his abdominal pain is a complication from his recent surgery. It's pertinent to note that he developed a fever postoperatively and he has been unable to tolerate a liquid diet, which could indicate an infectious process or complication from the surgery. Other alternate assumptions could be gastric issues due to his beer consumption, appendicitis, or even peptic ulcer disease, but these may not be clinically prioritized as they aren’t typical in the postoperative course. A ruptured abdominal aortic aneurysm repair has potential complications such as graft infection, graft thrombosis, or bowel obstruction, which should be considered. CLINICAL REASONING: Given that he doesn't have shortness of breath or chest pain, cardiac abnormalities are less likely, which reduces the likelihood of the pain being related to his previous myocardial infarction. His high leukocyte count could be indicative of an infection, likely due to a complication post-surgery, as it's typically rare for gastrointestinal disorders to cause such a significant leukocyte count increase without other symptoms. The lack of guarding or rebound tenderness lowers the possibility of peritonitis, but does not rule out intra-abdominal complications. CONCLUDING: In conclusion, given that the patient experienced a temperature, was unable to eat, and now has severe abdominal pain, he is likely dealing with something more severe than a simple gastric issue or stomach bug. The most likely cause is a complication from his recent surgery, such as graft infection, graft thrombosis, or bowel obstruction. RESPONSE: Thus, to evaluate the nature and confirm the location of the complication, an Ultrasonography of the abdomen (D) could offer the most immediately useful information. It's noninvasive, safe, readily available, and can also help to visualize any potential fluid collection, abscess formation, or identify if the graft or aneurysm has been compromised.

**AI_B:** (1) RESTATING THE QUESTION: The question presents a case study of a 67-year-old man in the postoperative phase of surgical repair of a ruptured abdominal aortic aneurysm. However, he has recently developed severe abdominal pain amid significant symptoms such as a low-grade fever. He is unable to tolerate a full liquid diet and has a medical history notable for hypertension, hypercholesterolemia, a previous myocardial infarction, along with habits of smoking and drinking. With notable physical examination and lab findings, we are tasked to identify the best additional diagnostic test for this patient. (2) PLANNING: To figure out what might be occurring, we need to look at several assumptions. An important assumption is his recent abdominal surgery. Also, the sudden onset of severe abdominal pain and a lingering low-grade fever should immediately raise suspicion for an infection or septic conditions. On the other hand, it's important to consider his risk factors, from surgery itself to his history of hypertension, hypercholesterolemia, and smoking. Each assumption corresponds to potential tests: arteriography could potentially reveal vascular problems stemming from the surgery; blood cultures would indicate systemic infection; an MRI or ultrasonography of the abdomen could potentially reveal post-surgical complications or other physical causes of his abdominal pain. Considering his signs, symptoms, and risk factors, it is reasonable to hypothesize that the infection is a likely cause. (3) CLINICAL REASONING: Connecting these hypotheses with clinical reasoning, the nature of the pain, as well as the patient's inability to tolerate a liquid diet and mild fever, point towards abdominal complications, likely infectious. Blood markers are showing mild left shift, indicating that the body is likely in an infection-fighting state. However, it's important to assess whether this infection is systemic or localized. (4) CONCLUDING: In order to choose the appropriate additional diagnostic study, it is crucial to identify the source of the infection which will dictate the patient's treatment. A systemic infection would potentially require more aggressive, broad treatment, while a localized infection could be managed with targeted therapy. As the abdominal pain suggests a potential intra-abdominal process, a study that visualizes this area directly without being too invasive might be most beneficial. (5) RESPONSE: Thus, option (D), 'Ultrasonography of the abdomen', would be the most appropriate additional diagnostic study in this case. It can detect a variety of post-surgical complications, such as an abscess or fluid collection, which might be causing the recent-onset abdominal pain and persistent fever and could also potentially indicate if there is a localized infection.

**AI_E:** The question is about deciding on an appropriate additional diagnostic study for a 67-year-old male who has recently undergone a surgical repair of a ruptured abdominal aortic aneurysm. The man is presenting with severe abdominal pain and other symptoms such as a low-grade fever that started 4 days ago. He has a medical history of hypertension, hypercholesterolemia, and a myocardial infarction 6 years ago. His current vital signs show mild fever and his physical examination reveals abdominal tenderness but no signs of guarding or rebound. His leukocyte count is elevated. Overall, the problem at hand requires distinguishing the cause of severe, postoperative abdominal pain in this patient with a significant cardiovascular history. One assumption could be that the abdominal pain is associated with his recent surgery. Given the potential severity of complications related to surgery, such as anastomotic leaks, hematoma, seroma or infections, this assumption takes high clinical priority. However, other assumptions worth considering would be unrelated problems, such as cholecystitis, peptic ulcers, or pancreatitis. Abdominal pain following aortic surgery could be due to various factors. A common postoperative complication is an anastomotic leak, which could lead to peritonitis evidenced by pain and tenderness in the abdomen. However, the patient does not have signs of peritonitis such as guarding or rebound. The elevated leukocyte count could suggest some kind of inflammatory response or infection. However, the examination findings do not specify the locus of the infection. Although it might be tempting to consider no further testing is needed given the mild fever and absence of peritonitis, the sudden onset and severity of the abdominal pain coupled with inability to tolerate diet suggests that further investigation is warranted. As such, we may need to explore additional diagnostic testing of the abdomen. Given the clinical picture, the priority is to identify the exact cause of his abdominal pain, and to do so non-invasively if possible. Among the options available, arteriography is invasive and is typically reserved for patients with suspected vascular problems. MRI, while comprehensive, can be time-consuming and may not be necessary in this case presentation. Blood cultures could provide additional information on possible septicemia but may not directly show the locus of infection or inflammation. Considering that an ultrasonography of the abdomen is a non-invasive method, and it can help us visualize possible fluid accumulation, inflammatory changes, and rule out unrelated problems such as cholelithiasis and pancreatitis, it seems like a sensible next step in this patient's care. Therefore, I would suggest that (D) Ultrasonography of the abdomen offers the most appropriate next diagnostic study for this patient. This decision could aid in determining any possible changes in the structure or fluids of the abdomen that could be leading to his acute abdominal pain.

**AI_C:** RESTATING THE QUESTION: Our patient, a 67-year-old who has been in the hospital for the postoperative management of a surgically repaired ruptured abdominal aortic aneurysm, is experiencing sudden severe abdominal pain. Postoperatively, he was doing fine until four days ago, when he developed a low-grade fever and difficulty tolerating a liquid diet. His past medical history is significant with notable for hypertension, hypercholesterolemia, and a prior myocardial infarction, and his current therapies include metoprolol, lovastatin, and daily aspirin. The patient is a heavy smoker, and he also consumes alcohol regularly. His physical examination revealed abdominal tenderness without evidence of peritonitis, and his laboratory findings indicate leukocytosis. Despite this, his electrolytes, urinalysis, and ECG show no acute abnormalities. The inquiry is about the most appropriate additional diagnostic study for this patient's case. PLANNING: Key assumptions made here include: 1) the abdominal pain is related to the surgical repair of the ruptured abdominal aortic aneurysm; 2) the fever and leukocytosis are signs of an underlying infection; and 3) his inability to tolerate a full liquid diet could be related to gastrointestinal complications or systemic illness. An alternate assumption challenges the first one, suggesting that the abdominal pain might not be directly related to the surgical repair but a complication such as infection, abscess, or ischemia. To evaluate this assumption, priority should be given to investigations that can directly visualize the abdominal structures and detect abnormalities in the postoperative region. CLINICAL REASONING: Potentially, the abdominal pain, fever, and leukocytosis could represent a local intra-abdominal infection such as an abscess, which can occur as a complication following abdominal surgery. The abdominal pain could also be due to ischemia in the gastrointestinal tract due to bowel hypoperfusion from the previous aortic aneurysm rupture or because of postoperative complications. Though routine abdominal imaging (MRI, Arteriography) might reveal these conditions, they are less sensitive and specific, particularly in the immediate postoperative period. A more sensitive and speedy investigation would be an ultrasound, which might detect localized fluid collections of an abscess or indications of bowel wall thickening indicative of ischemia. CONCLUDING: Given the recent surgery, the new onset of abdominal pain, the fever, the leukocytosis, and the patient's intolerance to a liquid diet, a complication due to surgery is strongly suspected. An abscess or ischemia secondary to altered blood flow post-abdominal aortic aneurysm surgery are both plausible. Immediate assessment with an imaging modality that can reliably identify these conditions is crucial. RESPONSE: Therefore, in this situation, (D) Ultrasonography of the abdomen would be the most appropriate additional diagnostic study for this patient. It is a relatively rapid, noninvasive, and reliable means of evaluating postoperative abdominal complications, such as an abscess or ischemia, which best fits the clinical picture here.

**Facilitator** The consensus among the AI responses is that the most appropriate additional diagnostic study for the 67-year-old man recovering from a surgical repair of a ruptured abdominal aortic aneurysm, who is now experiencing severe abdominal pain, is Ultrasonography of the abdomen (D). This conclusion is based on several key assumptions and clinical reasoning. The primary assumption is that the patient's abdominal pain is a complication from his recent surgery. The development of a fever postoperatively and his inability to tolerate a liquid diet could indicate an infectious process or complication from the surgery. His high leukocyte count could be indicative of an infection, likely due to a complication post-surgery. Given that he doesn't have shortness of breath or chest pain, cardiac abnormalities are less likely, which reduces the likelihood of the pain being related to his previous myocardial infarction. The lack of guarding or rebound tenderness lowers the possibility of peritonitis, but does not rule out intra-abdominal complications. The most likely cause is a complication from his recent surgery, such as graft infection, graft thrombosis, or bowel obstruction. Therefore, to evaluate the nature and confirm the location of the complication, an Ultrasonography of the abdomen could offer the most immediately useful information. It's noninvasive, safe, readily available, and can also help to visualize any potential fluid collection, abscess formation, or identify if the graft or aneurysm has been compromised.
